# A New *Casjensviridae* Bacteriophage Isolated from Hospital Sewage for Inactivation of Biofilms of Carbapenem Resistant *Klebsiella pneumoniae* Clinical Isolates

**DOI:** 10.3390/pharmaceutics16070904

**Published:** 2024-07-05

**Authors:** Sambuddha Chakraborty, Anusha Rohit, S. Jaya Prasanthi, Ashwini Chauhan

**Affiliations:** 1 Department of Microbiology, Tripura University, Suryamaninagar 799022, India; 2 Department of Microbiology, University of Delhi South Campus, Benito Jaurez Marg, New Delhi 110021, India; 3 Madras Medical Mission Hospital, Chennai 600037, India

**Keywords:** antimicrobial resistance, biofilm bacteriophage, *K. pneumoniae*, alternative to antibiotics, antibiofilm strategy

## Abstract

*Klebsiella pneumoniae*, a member of the ESKAPE pathogen group, is a prominent cause of hospital-acquired infections. The WHO has recognized carbapenem-resistant *K. pneumoniae* as a critical-one priority pathogen. These resilient superbugs have the ability to form biofilms and present a significant global threat. In the present study, we isolated and characterized a bacteriophage SAKp02, from hospital sewage, infectious to carbapenem-resistant *K. pneumoniae* patient isolates. SAKp02 could infect 43 of 72 clinical isolates, indicating a broad host spectrum. Whole genome analysis classified SAKp02 within the family *Casjensviridae*, with a 59,343 bp genome encoding 82 ORFs. Comparative genomic analysis revealed significant differences between SAKp02 and its closest viruses, indicating a distinct genetic makeup positioning it as a novel phage strain within the lineage. The SAKp02 genome comprises bacteriolytic enzymes, including holin, endolysin, and phage depolymerase, crucial for bacterial lysis and biofilm disruption. It reduced biofilm biomass by over threefold compared to the control and eradicated 99% of viable cells within a 4 h treatment period. Scanning electron microscopy corroborated the ability of the phage to dismantle biofilm matrices and lyse bacterial cells. Safe and effective treatments are warranted, and hence, the fully characterized lytic phages with therapeutic potential against drug-resistant clinical isolates of bacteria are needed. Our study is the first to report the antibacterial and antibiofilm activity of *Casjensviridae* phages, and our discovery of a novel *K. pneumoniae* phage broadens the arsenal against the bacteria.

## 1. Introduction

*Klebsiella pneumoniae* is an opportunistic oropharyngeal pathogen associated with nosocomial and community-based infections [1,2,3]. It belongs to the Enterobacteriaceae family, and the WHO has listed carbapenem-resistant *K. pneumoniae* as a priority one critical pathogen group [4]. Pathogenic *K. pneumoniae* can be categorized into three groups: healthcare-associated opportunistic or classic *K. pneumoniae* (cKp), a community-acquired infection causing hypervirulent *K. pneumoniae* (hyKp), and the global threat superbugs, i.e., multi-drug resistant (MDR) *K. pneumoniae* [5]. Studies showed that the MDR strains possess a greater risk of infection than the hyKp strains, as the MDR strains are more likely to acquire virulence compared to the propensity of hyKp strains to acquire drug resistance genes [6,7]. There are several contributory factors attributed to the pathogenicity of *K. pneumoniae*, such as capsule lipopolysaccharides and polysaccharides that help in evading the host immune system by hiding the bacteria from phagocytosis, fimbrial adhesin proteins that help in the adhesion of the bacterial cell to host tissues, and importantly, the ability to form biofilms on tissue or device surfaces [8,9,10]. *Klebsiella pneumoniae* demonstrates remarkable proficiency in biofilm formation, readily adhering to implanted medical devices and various host tissues [11,12]. Moreover, a correlation has been identified between the presence of antibiotic resistance genes and the biofilm formation ability of the bacterial strains. A study conducted by Yang et al. underscored a significant correlation between the presence of extended-spectrum beta-lactamase (ESBL) in clinical *K. pneumoniae* isolates and their propensity for biofilm formation [13]. Further exacerbating the clinical challenge, research by Anderl et al. revealed the inefficacy of antibiotics in disrupting *K. pneumoniae* biofilms. Even following prolonged exposure, ampicillin and ciprofloxacin exhibited a negligible impact on biofilm integrity [14]. Particularly concerning was the inability of ampicillin to penetrate biofilms harboring beta-lactamase-producing *K. pneumoniae* strains. The escalating emergence of antibiotic resistance coupled with the persistent resistance of bacterial biofilms to conventional antibiotic treatment underscores the urgent need for alternative therapeutic strategies. With the dearth of novel antibiotics in development pipelines, researchers are compelled to explore non-traditional avenues to combat *K. pneumoniae* infections effectively.

Bacteriophage, being the nature-built dedicated scavenger of bacteria, has gained a resurgent interest as an alternative approach to control bacterial infection. Phage discovery predates antibiotics by 13 years; however, antibiotics garnered widespread attention thereafter, owing to their potent killing activity and the subsequent surge in new antibiotic development. Over the past century, escalating drug resistance-associated disease burdens have spurred intensified research into phages as therapeutic agents. Bacteriophages emerge as indispensable antibacterial tools due to their ability to co-evolve with bacteria, ensuring that bacteria cannot evade phage, a limitation often encountered with antibiotics [15]. Studies indicate that developing resistance to phages may incur fitness costs to bacteria and reverse antibiotic resistance, potentially prolonging the efficacy of existing antibiotics [16,17]. Furthermore, bacteriophages possess inherent genetic mechanisms enabling them to hijack bacterial hosts, thus exhibiting innate bacteriolytic capabilities crucial for parasitizing bacteria and propagating within their populations. Moreover, bacteriophages exhibit the ability to penetrate and disrupt biofilms—a feature extensively documented in various studies [18]. However, several significant challenges confront phage therapy, foremost among them being the high specificity of phages to particular bacterial strains. Continuous efforts in isolating and characterizing novel phages remain imperative. Additionally, numerous parameters influencing the interaction between phages and bacteria, as well as factors contributing to the resurgence of phage resistance, necessitate ongoing scientific inquiry for a comprehensive understanding of these dynamics. In this study, we successfully isolated a lytic bacteriophage capable of targeting a multidrug-resistant clinical strain of *K. pneumoniae* from hospital sewage water. We extensively examined its bacteriolytic activity, host range, and ability to combat biofilms. The isolated phage was thoroughly characterized, including its efficiency in host cell attachment, the time a single phage takes to complete an infection (latency period), the number of progenies produced during the replication cycle (burst size), and the stability of the isolated phage. Additionally, we performed transmission electron microscopy to gain insight into the phage’s morphology and conducted a whole genome sequence analysis to understand its genetic composition. Moreover, an alternative to the antibiotic treatment approach is becoming a priority in combating drug-resistant bacterial infections, necessitating fully characterized lytic phages with therapeutic potential. For the first time, our study highlights the antibacterial and antibiofilm properties of *Casjensviridae* phages, showcasing their capability against resistant clinical isolates. Notably, our discovery of a novel *Klebsiella pneumoniae* phage significantly expands the arsenal available to target these extremely drug-resistant bacteria.

## 2. Materials and Methods

### 2.1. Bacterial Strains and Growth Conditions

The extensively drug-resistant (XDR) *Klebsiella pneumoniae* isolates procured from Madras Medical Mission Hospital (MMMH) Chennai, India, were cultivated at 37 °C in Luria-Bertani Broth (LB, Himedia, Thane, India, (M1245)) under shaking at 150 rpm until otherwise mentioned. To determine viable cell counts, serially diluted cultures were plated on sterile LB agar plates and incubated overnight at 37 °C. The colony forming units /mL were estimated. All the experiments were done in triplicates.

### 2.2. Isolation and Purification of Bacteriophage

Bacteriophage was isolated from the sewage water collected from the Madras Medical Mission Hospital (MMMH) Chennai, India, as described by Oliveira et al. with slight modifications [19]. Briefly, 5 mL LB was mixed with an equal volume of hospital sewage and 5 mL of mid-log phase XDR *K. pneumoniae* clinical isolate B3768 strain, grown to OD600~0.3–0.6. The mixture was incubated overnight at 37 °C shaking at 150 rpm. The mixture was centrifuged at 12,000× *g* for 10 min at room temperature (RT). Spot test was done for preliminary confirmation of the presence of lytic bacteriophage in the supernatant, where 10 µL of the supernatant was dropped on the top of the lawn of the bacterial isolate of interest and allowed to dry. The plate was incubated at 37 °C overnight. A clear zone of lysis was considered as positive bacteriolytic activity. The purification of a single phage was carried out using a double agar overlay technique by Kropinski et al. with slight modifications [20]. Briefly, a tenfold serial dilution of the supernatant was prepared. 100 µL of diluted supernatant was inoculated in 500 µL of an overnight culture of *K. pneumoniae*, and the whole mixture was added into 3 mL of 0.5% soft agar (Himedia India GRM026) and poured over 1.5% nutrient agar (NA) plate and incubated overnight at 37 °C. The next day, a single plaque was picked and inoculated in early log phase culture and kept for overnight incubation. The suspension was centrifuged at 12,000× *g* for 10 min at RT, and a double agar overlay technique was performed with the supernatant. The procedure was repeated thrice to get a single purified phage. Finally, the supernatant was treated with chloroform in a 1: 10 ratio for 15 min, after chloroform treatment the suspension was centrifuged and filtered through a 0.22 μm syringe driven filter (Himedia India TPP-99722) and stored at 4 °C.

### 2.3. Host Adsorption Assay

Adsorption assays were performed according to the procedure described by Mizuno et al. with slight modifications [21]. Briefly, 6.0 × 10^7^ cells/mL of *K. pneumoniae* B3768 strain was mixed with 6.0 × 10^6^ SAKp02 phage particles with a Multiplicity of Infection (MOI) of 0.1 in different microcentrifuge tubes and incubated at 37 °C in static condition for 30 min. At 5 min intervals, microcentrifuge tubes were removed and centrifuged for 4 min at 12,000× *g*, and unabsorbed phages present in the supernatant were determined by the double agar overlay technique.

### 2.4. Determination of Phage Life Cycle by One Step Growth Curve

A one-step growth curve to determine the latency and burst size of the phage was performed as described by Amarillas et al. with slight modifications [22]. *K. pneumoniae* B3768 strain was grown to a mid-exponential phase (OD: 0.6). Bacteriophage SAKp02 was mixed with an MOI 0.1 and kept for 10 min (according to previously evaluated adsorption time) at 37 °C in static condition. After incubation, the phage–bacteria suspension was centrifuged at 12,000× *g* for 10 min, and the supernatant containing unabsorbed phages was discarded. The pellet was resuspended in 10 mL of LB and incubated at shaking for 150 rpm for 2 h. 100 µL Samples were collected at every 20-min interval, and titration was done to determine the phage number.

### 2.5. Host Range of the Bacteriophage

A spot test was performed to evaluate the host spectrum of SAKp02. Lawn was prepared on the LB NA plates with the bacterial strains using mid-log phase cultures. Subsequently, 2.5 µL of purified phage SAKp02 was spotted on the lawn. The spots were allowed to dry, and the plates were incubated overnight at 37 °C. The appearance of clear lysis zones indicated susceptible bacterial strains, whereas the absence of plaques indicated phage-immune strains.

### 2.6. Assessment of Bacteriolytic Activity

A bacterial challenge test was performed to evaluate bacteriolytic activity with different MOIs. Briefly, the log phase culture of *K. pneumoniae* B3768 strain was grown up to exponential phase (OD: 0.600) (Bacterial cell number against particular OD was predetermined to adjust the MOI). The culture was centrifuged at 12,000× *g* for 10 min, and the supernatant was discarded. The pallet was resuspended in fresh medium and aliquoted into microcentrifuge tubes. Bacteriophage was added according to MOI 0.5, 1, 10, and 100. Then, 200 µL of phage–bacteria mixture of each MOI was dispensed in 96 well microtiter plates in triplicates. The plates were placed in a BioteK Epoch-2 microtiter plate reader (Agilent Technologies, Santa Clara, CA, USA) for 12 h. The OD of each well was taken at 20 min intervals.

### 2.7. Phage Stability

Phage stability was determined according to the method described by Sarkar et al. with slight modification [23]. Solvent Stability: 1 × 10^9^/mL Phage particles were mixed with PBS, chloroform, and 0.9% saline in equal proportion and incubated at 37 °C, 150 rpm for 1 h. Post incubation, the suspension was centrifuged at 12,000× *g* for 10 min, and the supernatant was used for phage titration. Thermal Stability: The exact amount of phage was kept at 25, 37, 55, 65, and 75 °C for 1 h. Titration was done post-incubation to assess the stability. pH Stability: The pH of LB broth was adjusted to 2–14. Phage lysate with a phage number 1 × 10^9^/mL was mixed equally and kept for 1 h at 37 °C. Titration was done post-incubation to assess the pH stability.

### 2.8. Transmission Electron Microscopy

On a clean piece of Parafilm, 100 µL of SAKp02 suspension with a concentration of ~10^9^ PFU/mL was dispensed. Prior to this, the Parafilm was cleaned by wiping it with 70% ethanol. Next, a 300 mesh Carbon Coated Copper grid (Electron Microscopy Sciences, Hatfield, PA, USA, EMS200-Cu) was gently positioned on the phage suspension sample and left undisturbed for 25 to 30 min. After the designated time, the grid was carefully lifted off from the samples. Excess liquid surrounding the grid was removed by gently dabbing it with filter paper, ensuring not to disturb the mesh. Subsequently, the grids were flipped over, and the sample side was placed down onto a droplet of 2% aqueous (wt/vol) phosphotungstic acid (Himedia, India, GRM398) solution (pH 6.8) for 3 min. To remove excess liquid, the grids were blotted dry using filter paper. They were then placed over another piece of filter paper and left to air-dry under a Petri plate, positioned upside down to prevent contact with the moist surface. After drying, the sample was imaged under Thermo Scientific™, Waltham, MA, USA, Talos L120C TEM (20–120 kV) at All India Institute of Medical Sciences, New Delhi, India.

### 2.9. DNA Extraction

A 50 mL crude bacteriophage sample was sent to Redcliffe Lifetech Pvt. Ltd., Noida, India Ltd., for DNA extraction and genome sequence by Illumina Hiseq platform. Phage DNA was exposed to RNase at a concentration of 3 μg/mL and DNase at a concentration of 1 μg/mL. This treatment occurred overnight at a temperature of 37 °C. Subsequently, the phage suspension was subjected to incubation at 80 °C for 15 min to deactivate the enzymes. Following the deactivation step, the purified phages were treated with proteinase K at a concentration of 50 μg/mL. This treatment took place at a temperature of 56 °C for 1 h. Additionally, SDS was present in the treatment at a concentration of 0.5%. Then it was mixed with an equal volume of a phenol/chloroform/isoamyl alcohol solution (25:24:1 ratio). The resulting mixture was then centrifuged at 13,000× *g* for 5 min at 4 °C. After centrifugation, the supernatant was mixed with an equal volume of isopropanol and stored at −20 °C for 1 h. Following this, another round of centrifugation took place. To wash the pellet, 1 mL of 75% ethanol was added and allowed to sit at room temperature for 1 min. Subsequently, the mixture underwent centrifugation at 12,000× *g* for 10 min at 4 °C. The resulting pellets were air-dried and then dissolved in 30 µL of ddH_2_O. Finally, they were stored at −20 °C for future use. Samples Concentration and Quality was estimated using Qubit 4.0 Fluorometer. All extracted DNA Samples were run and checked on 0.8% Agarose Gel made in 1X TAE Buffer.

### 2.10. Bacteriophage Whole Genome Sequencing, Annotation and Bioinformatics

Phage whole genome sequencing was performed in the Illumina Hiseq platform. Denovo genome assembly was done by the St. Petersburg genome assembler (SPAdes). It is an assembly toolkit containing various assembly pipelines. SPAdes support paired-end reads, mate-pairs, and unpaired reads. It can take as input several paired-end and mate-pair libraries simultaneously. SPAdes was initially designed for small genomes. It was tested on bacterial (both single-cell MDA and standard isolates), fungal, and other small genomes. We assembled the data and extracted the phage sequence using phaster and annotated the same using Prokka (1.14.6). Quality control was done by fastp (0.20.1) The Fastp tool is designed to provide fast all-in-one pre-processing for FastQ files. This tool is developed in C++ with multithreading supported to afford high performance. Quality control and pre-processing of FASTQ files are essential to providing clean data for downstream analysis (https://github.com/OpenGene/fastp, accessed on 10 May 2024). The phage sequence was annotated using the prokka tool. Prokka is a command line tool for the rapid annotation of bacterial, archaeal, and viral genomes. Further annotation was performed and a genome map was constructed using RAST version 2 (https://rast.nmpdr.org/rast.cgi, accessed on 10 May 2024), BLASTp server (https://blast.ncbi.nlm.nih.gov/Blast.cgi?PAGE=Proteins, accessed on 10 May 2024), PHASTER (https://phaster.ca/, accessed on 10 May 2024) PhaGAA (http://phage.xialab.info/predict, accessed on 10 May 2024), genomevx (http://wolfe.ucd.ie/GenomeVx/, accessed on 10 May 2024), and Geneious Prime^®^ 2023.0.4; for t-RNA in the genome, tRNAscan-SE v. 2.0 (http://lowelab.ucsc.edu/tRNAscan-SE/, accessed on 10 May 2024) was used. Phylogenetic tree analysis was performed in MEGA11 software (https://www.megasoftware.net/, accessed on 10 May 2024) based on the whole genome sequence as well as the large terminase subunit. EasyFigure (https://mjsull.github.io/Easyfig/files.html, accessed on 10 May 2024) was used to compare the related viral genomes. An essential criterion for phages to be used as therapeutics is that they should not be carrying any drug-resistance genes or virulent factors. We have used ResFinder 4.1 (https://cge.food.dtu.dk/services/ResFinder/, accessed on 10 May 2024) developed by Centre for Genomic Epidemiology to find drug resistance genes in a genome, and presence of virulent factor was examined in Virulence Factor Database (VFDB) (http://www.mgc.ac.cn/VFs/search_VFs.htm, accessed on 10 May 2024).

### 2.11. Assessment of Anti-Biofilm Activity of SAKp02

The biofilm inhibition assays were conducted according to the method outlined by Chauhan et al. 2014, with slight modifications [24]. Overnight cultures of the *K. pneumoniae* B3768 strain were diluted 1:100 in fresh media and inoculated into a 96-well microtiter plate. The plate was then incubated at 37 °C without shaking for 24 h to allow the formation of mature biofilms. Subsequently, the planktonic bacteria were gently removed by pipetting, and the wells were rinsed three times with PBS. Next, the 24-h-old mature biofilms were treated with SAKp02. A 100μL solution of the phage suspension was added to the wells at MOI 1 and left in the wells for specific time intervals (4 h, 8 h, and 12 h) at 37 °C under static conditions. Control wells were left untreated and inoculated with PBS. After the respective treatment period, the phage suspension was removed, and the wells were rinsed three times with PBS. The experiment was conducted in two sets with four replicates each. One set was used for viable cell count, and the other set was used for biomass estimation using crystal violet (CV) staining. For the viable cell count, the wells were vigorously scraped to remove any remaining biofilm, and the resulting mixture was homogenized with PBS. Viable cell count was determined by plating serial dilutions on LB agar medium and incubating the plates at 37 °C. For the crystal violet assay, after washing the wells with PBS following the treatment, the wells were stained with 0.1% CV. After a 15-min incubation period, the stain was carefully removed, and the wells were rinsed with PBS to remove any excess stain. To dissolve the CV stain adhered to the wells, a solution of acetone and ethanol was added. The solution was mixed thoroughly by pipetting and then transferred to a separate flat-bottom microtiter plate for absorbance measurement at a wavelength of 580 nm.

### 2.12. Scanning Electron Microscopy

Scanning electron microscopy (SEM) of the biofilm was performed as described by Chauhan et al. 2016 with slight modification [25]. Briefly, one cm^2^ glass coverslips were placed in individual wells of 6-well plates, and an O/N culture of *K. pneumoniae* B3768 diluted to 1:100 was inoculated in 6-well plates to allow the biofilms to form on the coverslips. The plate was incubated at 37 °C under static conditions for 24 h. Post incubation, the coverslips were washed thrice with PBS to remove any loosely adhered bacteria. The biofilms were treated with phage SAKp02 and incubated for a required time at 37 °C under static conditions. The untreated wells were taken as controls. Coverslips from the wells were taken out and dipped in the fixative solution (2.5% glutaraldehyde solution in PBS) overnight. After overnight fixation, the slides were washed with PBS and dehydrated with 10, 20, 30, 40, 50, 60, 70, 80, 90, and 100% ethanol for 10 min each. The slides were air-dried, gold-sputtered, and observed under Sigma 300, Carl Zeiss Field Emission Scanning Electron Microscope, Tripura University, Agartala, India.

### 2.13. Statistical Analysis

The results presented in this study are based on a minimum of three independent experiments, ensuring reproducibility. To determine statistical differences, an unpaired t test was conducted using GraphPad Prism version 8.0. Statistical significance was defined as *p*-values less than 0.05.

## 3. Results

### 3.1. Phage SAKp02 Forms Depolymerase-Induced Bull’s Eye Plaques with a Short Adsorption Time and a 20-Minute Latency

**Figure 1:**
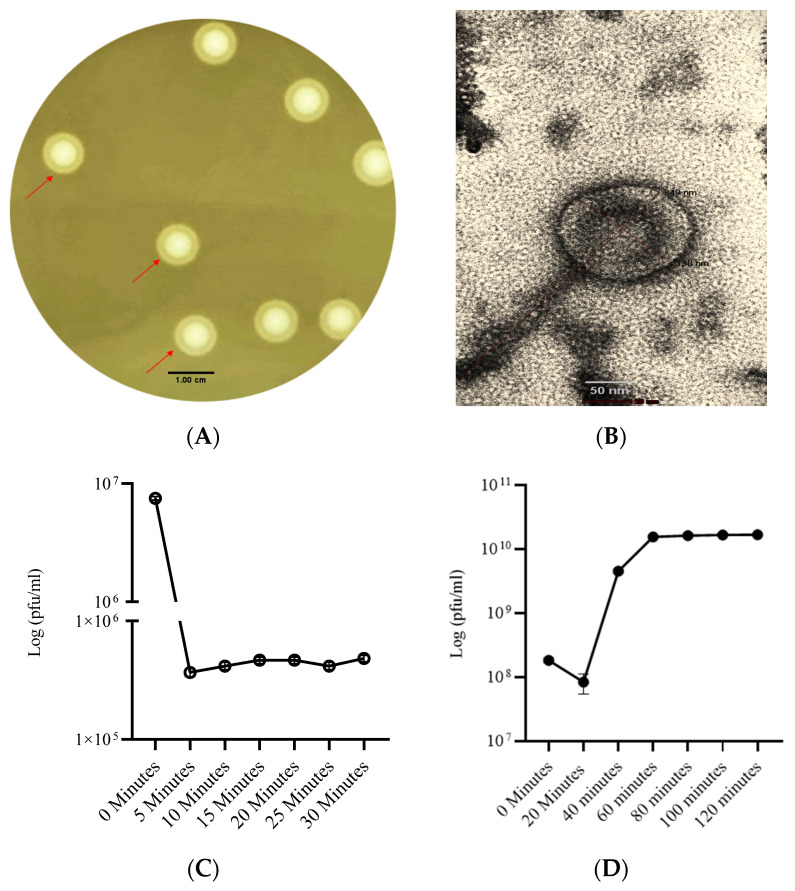
Plaque and structural morphology of phage SAKp02: (**A**) Plaque morphology of phage SAKp02 on the lawn of *K. pneumoniae* B3768 strain. Depolymerase activity is marked with an arrow; (**B**) TEM Image of phage. Adsorption rate and time of SAKp02 and single step growth curve: (**C**) SAKp02 took 5 min to adsorb to the host with a rate of 95% of total phage particles; (**D**) Phage SAKp02 has shown a 20 min latency period and a burst size of ~202 phage particles per host cell.

Bacteriophage SAKp02 was isolated against the *K. pneumoniae* XDR clinical strain B3768 from the hospital sewage of MMMH. The phage could produce clear, medium-sized plaque approximately 1 cm in diameter with depolymerase activity. A typical bull’s eye plaque morphology in the bacterial lawn was observed (Figure 1A). The TEM analysis revealed that the length of phage SAKp02 is 349 nm with a capsid size of 138 nm. The tail of SAKp02 is 211 nm long, and the phage consists of a neck, a contractile tail, and a central tube (Figure 1B). The adsorption assay clarified the time required for a virus to attach to the host receptor, which is a crucial parameter for analyzing the infectivity of the virus. The average adsorption time necessary for a phage to initiate an infection has been reported as 10 to 15 min. However, phage SAKp02 showed a relatively shorter adsorption time of 5 min (Figure 1C), and 95% of the phages could adsorb to the host. One-step growth curve data indicated that the phage SAKp02 has an eclipse period of approximately 20 min and a rise period of 40 min with a burst size of approximately 202 phages per bacterial cell (Figure 1D).

### 3.2. Phage SAKp02 Is Strongly Bacteriolytic with a Broad Host Range

**Figure 2:**
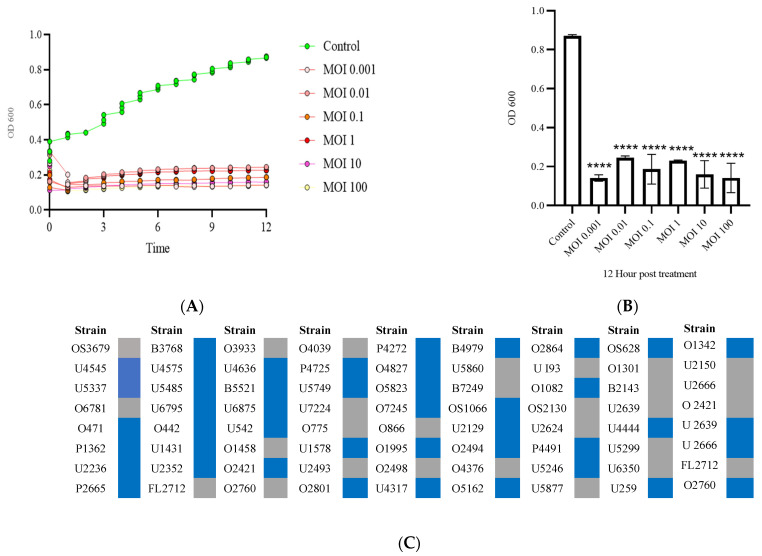
Killing kinetics and host range of phage SAKp02. (**A**) Phage SAKp02 could restrict the growth of clinical strain B3768 in comparison to the control (Bacteria + LB) at different MOI. (**B**) Endpoint OD of the control and phage treatment with different MOIs after 12 h post-phage treatment. (**C**) Phage SAKp02 could infect 43 *K. pneumoniae* clinical isolates among 72 tested strains. Data represented are means ± standard error of the mean of at least 3 independent experiments. **** *p* < 0.0001.

To evaluate the lytic activity of bacteriophage SAKp02, a bacterial challenge test was performed with different MOIs (MOI; 0.001, 0.01, 0.1,1,10, and 100). The assessment of bacteriolytic activity is crucial to determine the nature of the phage, whether the phage is lytic or lysogenic. It is reported that the temperate phage enters into lysogeny soon after the initiation of infection and does not restrict the growth of bacteria for longer. Phage SAKp02 could control the bacterial growth in different MOIs for 12 h (Figure 2A,B). Clinical isolates of *K. pneumoniae* isolated from MMM hospital were used to evaluate the host range of SAKp02, and a total of 72 clinical isolates were tested for the host range. Among the clinical isolates, phage SAKp02 could infect ~60% of clinical isolates with a clear lysis zone (Figure 2C).

### 3.3. Phage SAKp02 Is Stable at Different pH, Temperature, and Solvent

**Figure 3:**
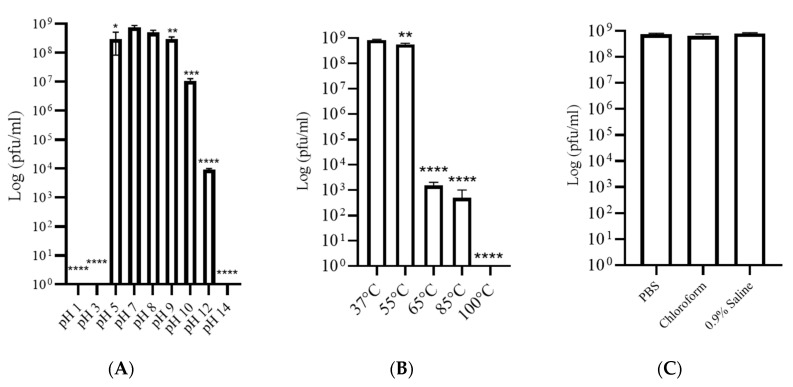
Stability studies of phage SAKp02; (**A**) At pH 1, 3, and 14, complete degradation of phage SAKp02 was observed. (**B**) At 65 °C, a significant~6-fold reduction was observed, and complete denaturation of phage particles at 100 °C was seen. (**C**) SAKp02 showed stability in PBS, chloroform, and 0.9% saline. Data represented are means ± standard error of the mean of at least 3 independent experiments. *p < 0.05, ** *p* < 0.005, *** *p* < 0.0005, **** *p* < 0.0001.

When considering a therapeutic approach, phage stability is a critical concern. Thermal stability, the effect of various pH on the virus, and stability with different solvents are also significant concerns. Hence, the impact of pH, temperature, PBS solution, 0.9% saline solution, chloroform, and ethyl alcohol were assessed. Most of the eukaryotic as well as prokaryotic viruses are most stable in the neutral pH; phage SAKp02 showed a similar pattern, and the most significant decrease in phage titer was observed from pH 12, and in pH 1, 3, and 14, no plaque was observed (Figure 3A). Phage SAKp02 showed stability up to 55 °C. However, a significant decrease in phage titer was observed at 65 °C and 85 °C, and at 100 °C, no plaque was observed (Figure 3B). Phage SAKp02 was adequately stable in PBS, 0.9% saline, and chloroform (Figure 3C).

### 3.4. SAKp02 Belongs to the Casjensviridae Family of Viruses

**Figure 4:**
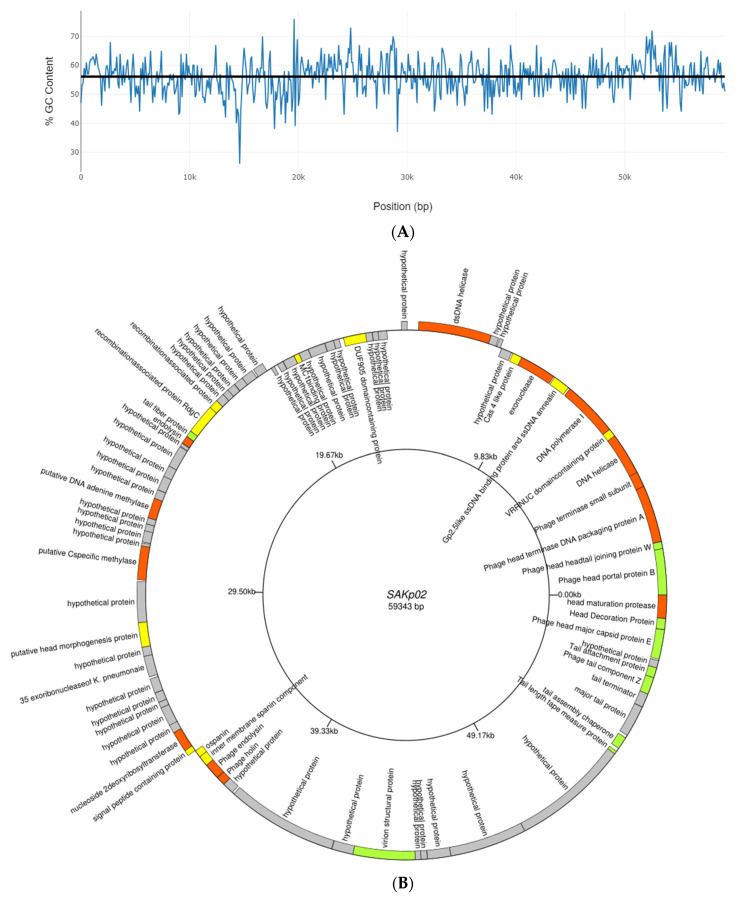

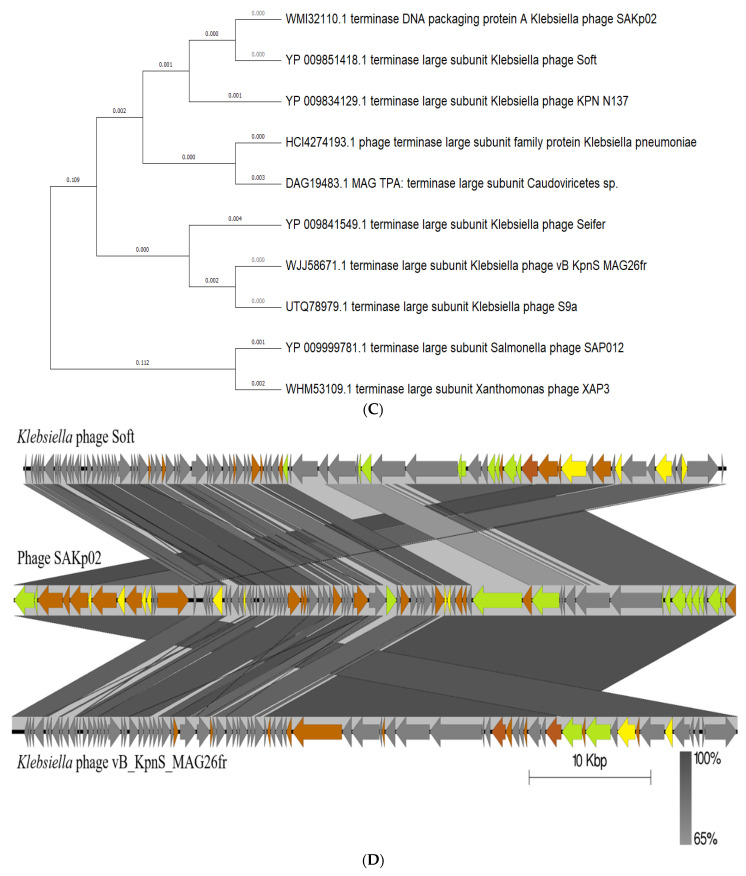

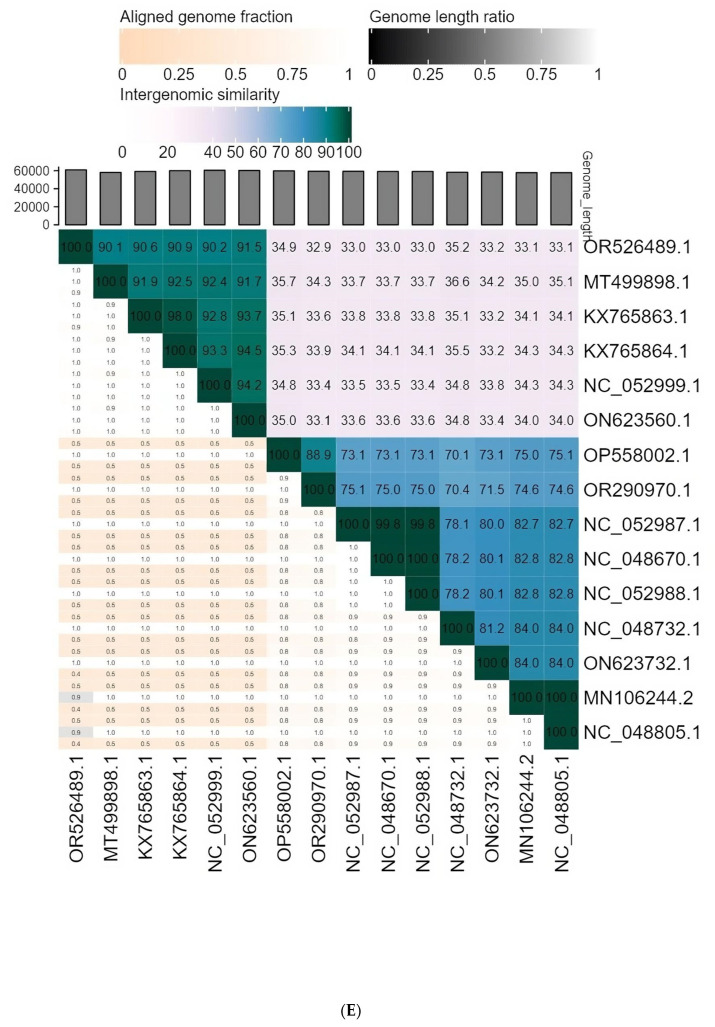
Overview of whole genome of phage SAKp02 and bioinformatic analysis. (**A**) The GC content and genome alignment of phage SAKp02 constructed by GC Content calculator. The blue skew indicates the GC content according to the nucleotide regions. Mean GC content is marked as a straight black line. Calculation shows that the GC content of SAKp02 is 56.1%. (**B**) The genome map of phage SAKp02 generated by the genomevx. ORFs coding for hypothetical proteins are denoted in grey. Genes with predicted functions are colored; orange-colored genes are indicated as phage enzyme associated with replication process at different stages of viral life cycle, green colored CDSs are denoted as structural proteins of the virus, and yellow colored CDSs are proteins associated with regulatory activities. (**C**) Neighbor-joining phylogenetic tree of terminase large subunits from bacteriophage SAKp02 and the related phages. Sequences were downloaded from NCBI. (**D**) EasyFigure was used to create a linear representation and comparative analysis, visualizing alignments phage Soft (top DNA), SAKp02 (middle DNA), and phage vB_KpnS_MAG26fr (bottom DNA). Arrows denote the transcription direction of predicted ORFs and are categorized as grey (hypothetical proteins), green (structural proteins), orange (viral enzymes), and yellow (regulatory proteins). Shaded regions indicate well-conserved segments with 65–100% amino acid similarity, with darker grey regions showing higher identity. The scale is in 10 kbp increments. (**E**) A heatmap generated by VIRIDIC illustrates the intergenomic similarities on the right half, using color-coding to provide a quick visual comparison of the 10 phage genomes most closely related to SAKp02 (NC-048805.1: Phage soft, OR290970.1: SAKp02, OP558002.1: *Klebsiella* phage vB_KpnS_MAG26fr).

The genome of phage SAKp02 is 59,343 base pairs (BP) long with a GC content of 56.1%, comprising 82 Open Reading Frame (ORF) and no tRNA genes (Figure 4A). Out of the total ORFs, 38 show significant homology with functional proteins, leaving 54% of the protein as hypothetical function; this suggests that a significant portion of the predicted proteins still need to be characterized, with their roles and functions in phage biology still being determined (Supplementary Table S1). The annotated genome map of phage SAKp02 is depicted in Figure 4B. The SAKp02 genome is compact, linear, and has only 6.1% intragenic space. The longest gene in SAKp02 is 4301 nucleotides, with its functional features yet to be identified. The smallest gene is 113 nucleotide long encoding tape measure protein, which facilitates the transit of the viral DNA in host cytoplasm during infection. The whole genome of SAKp02 shows a significant similarity with *Klebsiella* phage vB_KpnS_MAG26fr, belonging to family *Casjensviridae* and the class *Caudoviricetes.* Phylogenetic analysis based on the terminase large subunit indicates a homology with *Klebsiella* phage Soft, also a member of *Casjensviridae* (Figure 4C). Comparative genomic analysis of phage SAKp02 with two *Klebsiella* phages showed high similarity (Figure 4D). A BLAST search revealed a high similarity in nucleotide percent identity between *Klebsiella* phage vB_KpnS_MAG26fr and phage SAKp02, while the phylogenetic analysis of the large terminase subunit indicated a closer similarity with Klebsiella phage Soft. VIRDIC analysis of intergenomic similarity of phage SAKp02 with *Klebsiella* phage soft and *Klebsiella* phage vB_KpnS_MAG26fr has shown a high similarity, indicating the clustering of the phages in the similar family; however, they are distinct novel phages (Figure 4E). We found the phage genome was without any antibiotic-resistant genes or virulent factors, which is ideal for therapeutic use. The genome of SAKp02 has been deposited to NCBI, accession number OR290970.1, https://blast.ncbi.nlm.nih.gov/Blast.cgi#alnHdr_2523361381, accessed on 10 May 2024.

### 3.5. SAKp02 Effectively Dismantles K. pneumoniae Biofilm and Lyse Biofilm Residing Bacteria

**Figure 5:**
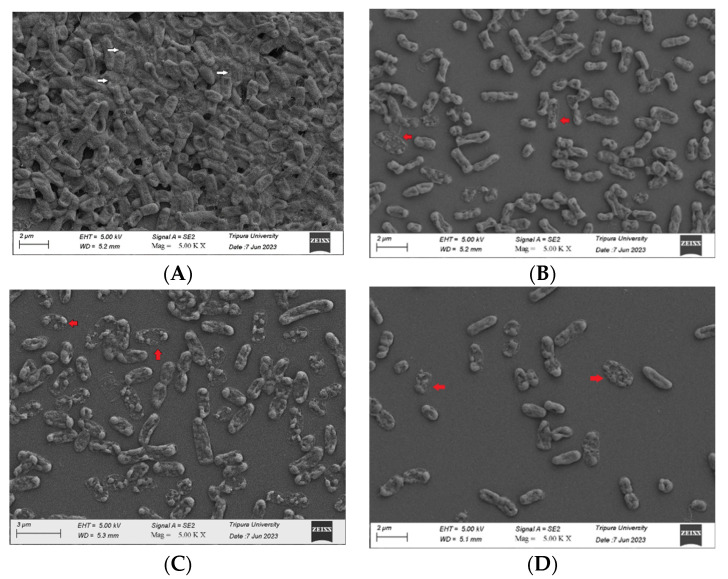
Assessment of antibiofilm activity of SAKp02. (**A**) The scanning electron microscopy (SEM) analysis (sample coating, gold sputter coating; mounting, carbon tape; accelerating voltage, 5.00 kV; working distance, 5.1–5.3 mm; magnification, 5.00 K X; imaging, secondary electron imaging; detector, secondary electron detector; contrast, topographic contrast) revealed that *K. pneumoniae* exhibited a compact bacterial community covered by an extracellular polymeric substance (EPS) layer (white arrows). This observation indicates strong biofilm formation by *K. pneumoniae* MDR clinical strains. (**B**) Four-hour treatment with SAKp02 could effectively degrade the EPS layers and reduce the density of the bacteria. (**C**,**D**). After 8 and 12 h of treatment with phage SAKp02, there was a noticeable degradation of the biofilm matrix and bacterial cell. Cellular debris (red arrow) on the slides indicates a lytic phage infection.

The scanning electron microscopy (SEM) analysis provided valuable insights into the impact of bacteriophage SAKp02 on 24-hour-old biofilm. The control slide displayed a well-established biofilm structure, characterized by densely packed bacteria coated with extracellular polymeric substances (EPS) (Figure 5A). However, exposure of the biofilm to SAKp02 resulted in significant disruption of the biofilm. The biofilms were visibly disintegrated, with degraded EPS and cell debris remaining on the coverslips. This visual observation indicated that bacteriophage SAKp02 had an eliminatory effect on the biofilm; it can break down the protective EPS matrix and lyse the biofilm-residing bacterial cells (Figure 5A–D).

### 3.6. SAKp02 Potentially Reduces Biofilm Biomass and Viable Bacterial Cell

**Figure 6:**
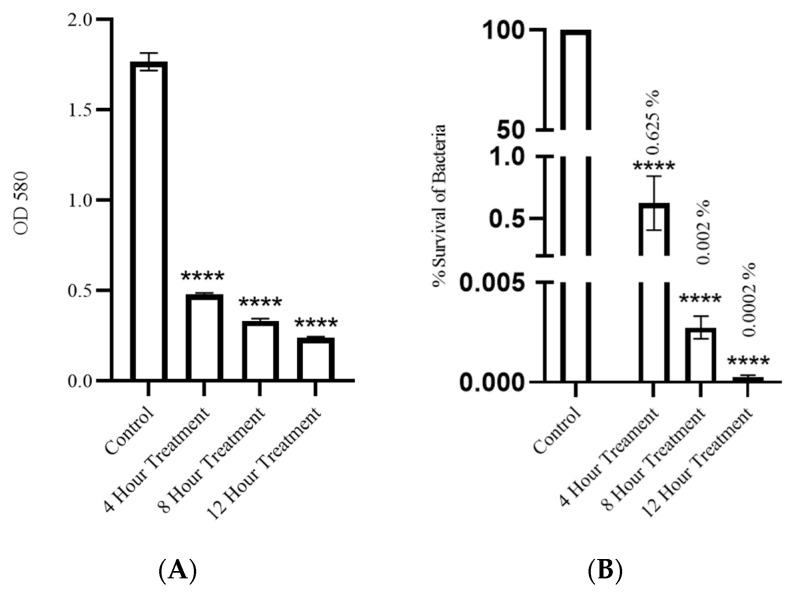
Evaluation of antibiofilm efficacy of SAKp02. (**A**) Crystal violet assay indicated an effective demolition of biofilm biomass after 4 h of phage treatment; 12 h of phage treatment showed the lowest biofilm biomass; a 7-fold decrease in OD compared to control is seen. (**B**) Effective lysis of biofilm viable cells was seen with SAKP02 infection; only <0.001 percent of bacteria survived after 12 h of phage treatment. Data represented are means ± standard error of the mean of at least 3 independent experiments. **** *p* <0.0001.

To evaluate the antibiofilm activity, a crystal violet assay was conducted to measure the optical density at 580 nm (OD580) of the solubilized crystal violet, which serves as an indicator of biofilm biomass. The results demonstrated that after SAKp02 treatment, a significant reduction in biofilm biomass was achieved. SAKp02. Twelve hours post-treatment with bacteriophage SAKp02, a substantial 7-fold decrease in optical density compared to the control indicated the effective reduction of biofilm formation by the phage (Figure 6A). To validate these findings, bacterial colony counting was performed before and after the introduction of bacteriophage. This quantitative analysis provided evidence of the impact of phage treatment on biofilm-residing bacterial cells. After 4 h of exposure to SAKp02, there was a 99.3% reduction in bacterial cells. However, the most pronounced reduction in viable bacterial cells in biofilm, amounting to a 99.999% reduction, was observed at 12 h post-infection with SAKp02 (Figure 6B). These results further confirmed the antibiofilm activity of bacteriophage SAKp02 and highlighted its effectiveness in reducing *K. pneumoniae* biofilm.

## 4. Discussion

Due to the high emergence of drug resistance, the treatment outcomes for *Klebsiella pneumoniae* have been extremely poor, prompting the World Health Organization (WHO) to designate this micro-organism as a “priority-one critical pathogen” for the research and development of new antibiotics and new alternative treatments in 2017 [26]. A follow-up report revealed the insufficiency of the development of newer antibiotics. Additionally, the ability of MDR *K. pneumoniae* to form biofilms further compounds the difficulties of treating patients [11,12]. Recent studies indicate that utilizing bacteriophages shows promise as an effective strategy against biofilms [27]. However, one of the major challenges in implementing phages as a clinical therapy is their host specificity, as most phages exhibit high specificity for their particular host strains; hence, it is crucial to isolate and fully characterize the bacteriophages, infectious against drug-resistant strains. Several studies have been conducted to isolate and characterize phages infectious to drug-resistant bacteria to strengthen the arsenal against the MDR strains [21,28,29]. However, a continuous flow of phage isolation and characterization is required; it will expand the phage library against AMR strains, which can be further utilized for therapeutic purposes. Moreover, the accumulation of experimental data will be useful for the progression of phage therapy [30,31]. This study isolated phage SAKp02, infectious against several drug-resistant clinical strains of *K. pneumoniae*, from hospital sewage water. A clear transparent plaque indicates the virulent activity of the phage SAKp02 against the respective clinical strains. The plaques developed a halo, a semi-transparent clearing zone encircling the transparent core lysis zone (Figure 1A). Halos are formed due to the diffusion of phage-encoded enzymes through the bacterial lawn. Halos appeared after 24 h of incubation. Halo formations are considered to be an indication of a strong depolymerase activity and are mostly the resultant of tail proteins [32,33]. The genomic analysis of SAKp02 identifies the tail fiber protein ORF36 (Supplementary Table S1) reported for the depolymerase activity, which supports the plaque formation pattern of SAKp02 on the bacterial lawn. It has been reported that the plaque size formed by a bacteriophage on a bacterial lawn depends on the phage’s burst size [34]. Our data corroborate this finding, as the burst size of SAKp02 is approximately 202 phages per bacterial cell, which is relatively large. Consequently, SAKp02 forms a considerably large plaque on the bacterial lawn. [22,35,36]. Electron microscopic image revealed the SAKp02 as a tailed phage (Figure 1B). Whole genome analysis revealed that the phages belong to the class of tailed bacteriophages, *Caudoviricetes* and the family *Casjensviridae* [37]. The adsorption rate, period, and time required to replicate and produce phage particles per host cell are important for phage infectivity. Since these two actions happen sequentially and are dependent on one other, the rate at which phages commence infection and the rate at which adsorption occurs are typically regarded as synonymous. [38,39]. The time required from adsorption to the bursting out of the progeny virus after one infection cycle is attributed as eclipse or latency period. We found that the phage SAKp02 is relatively quick in being adsorbed to the host compared to the average adsorption period of reported phages [40]. SAKp02 could adsorb to the bacterial host in as soon as 5 min (Figure 1C). To complete the replication cycle, phage SAKp02 took 40 min (Figure 1D). In approximately 40 min, phage SAKp02 could produce approximately 202 phage particles per bacterial cell. The bacteriolytic activity of the phages is the most crucial parameter to consider when using the respective phage in therapy. Several studies have reported the development of phage-resistant post-phage treatment within hours [41]. Resistance can be developed in several ways: modulation in host receptor, thus inhibiting the phage attachment; blocking of phage DNA from entering inside bacterial cells; or nuclease mediated [42]. However, the development of phage resistance can interfere with the efficacy of phage therapy. Here, we have evaluated the bacteriolytic efficacy of phage SAKp02 with different MOIs (Figure 2A,B). Several studies have reported MOI-dependent lytic activity and phage resistance resurgence. It is observed that at high MOI, the selection pressure on the bacterial population is intense, which can lead to the rapid emergence of resistant mutants. These resistant strains can proliferate once the susceptible bacteria are lysed, leading to a resurgence of the infection [43]. However, with a different range of MOIs, phage SAKp02 could control bacterial growth for 12 h, and the emergence of phage resistance has not been reported for any of the MOIs. This indicates a potent bacteriolytic activity of SAKp02. The host range of the phage is also an important factor to be included in the arsenal of phage therapy. SAKp02 could infect several drug-resistant clinical strains (Figure 2C), possibly indicating a common cell membrane protein required for the phage attachment. More studies are required to explore this possibility, which is one of the major concerns of phage therapy [44]. Host range was checked by the spot test assay. Spot tests can sometimes yield false positives due to bacterial cell lysis without actual phage infection. This may occur because a high number of phage particles adsorb to the bacterial cells and cause “lysis from without” or due to the presence of residual endolysins in the phage suspension [45]. However, the spot tests were repeated several times to confirm the host range, and all the replicates indicated a similar spectrum of host range. Residual endolysin-mediated killing could cause deviation in the findings. The stability study revealed that the phage is substantially stable at 37 °C and range of pH (Figure 3A,B). One of the major concerns of any therapeutics containing live biological content is pharmaceutical excipients. Pharmaceutical excipients can also impact the stability of several vaccines. Phages, being the virus, need to be administrated and formulated in a constituent; thus, solvent stability is one of the important parameters regarding the longevity of the phage as a therapeutic constituent. SAKp02 has shown stability in PBS and 0.9% saline, the most common solvents used in experiments, as well as 0.9% saline, which is the most commonly used intravenous fluid given to patients. Genomic analysis revealed that the phage SAKp02 belongs to the family *Casjensviridae* and the genus *Yonseivirus*, one of the less-studied bacteriophage families. Tail-like phages have evolved a range of strategies and enzymes to make their way through the several layers of bacterial cell membranes. Their primary objective is to deliver genetic material into the host cell’s cytoplasm, where it will multiply. They discharge the virion progeny after the reproductive cycle is finished. There is an important distinction between these two critical stages of the phage infection cycle: effective virion progeny release often requires host cell damage or lysis, whereas viral entrance must not impact cell viability [46]. The whole genome sequences have identified several important proteins associated with phage lysis: holins, endolysin, tail fiber (spike) protein, or depolymerase (Figure 4B) (Supplementary Table S1). Experimental data on the bacteriolytic activity has shown the efficient lytic activity of the phage SAKp02, indicating the efficient activity of the lytic enzymes. SAKp02 shows high nucleotide homogeneity with Klebsiella phage vB_KpnS_MAG26fr and Klebsiella phage Soft SAKp02. EasyFigure was used to analyse the genomic similarities of phage SAKp02 with previously reported *Klebsiella* bacteriophages Vb_KpnS_MAG26fr and Soft. BLAST comparison between three phages indicates a high nucleotide identity of several CDS regions ranging from as low as 65% to complete homogeneity. However, despite a high nucleotide identity, we can observe a dynamic genomic rearrangement between these genomes (Figure 4D), indicating strong evolutionary footsteps. Data on *Casjensviridae* phages are limited; hence, comparisons about bacteriolytic or antibiofilm properties could not be made. Moreover, this is the first extensive study on *Casjensviridae* phages. We found that phage SAKp02 does not carry the genes associated with pathogenicity or drug resistance; hence, it is safe for therapeutic use. Numerous factors that may have an impact on controlling bacterial biofilm were found through correlation studies. More significant biofilm reduction was found to be achieved by higher phage concentrations. Furthermore, phages with larger burst sizes and shorter latent periods were found to be the best options for in vitro biofilm reduction [47]. SAKp02 could significantly reduce the biofilm formed by drug-resistant *K. pneumoniae* strains after 4 h of treatment AT MOI 1. The most crucial component of the biofilm is EPS; in the control slide of scanning microscopic image (Figure 5A), we could observe that the bacterial cells are densely packed and embedded in the side EPS layer. Certain phages like SAKp02 can encode extracellular polysaccharide depolymerases, which selectively break down the EPS and easily enter the deeper layers of the biofilm architecture [27,48]. Right after 4 h of SAKp02 treatment, a reduction in the EPS layer could be seen (Figure 5B). The SEM data reflect the data of biofilm biomass reduction and viable bacterial cell reduction (Figure 6A,B). After 4 h of treatment, approximately a 3-fold reduction in OD could be seen. Several studies have documented a reduction in biofilm biomass and viable cell count [49,50,51]. However, prolonged exposure to a single phage strain has also been associated with the emergence of drug resistance [52]. Extensive research on the emergence of phage resistance and the impact of phage resistance on bacterial fitness is required. Several researchers recommend a combination of antibiotics with phages to reduce the chance of developing phage and antibiotic resistance. The application of phage cocktails could be another approach to minimize phage resistance. Considering the rapidly emerging drug resistance, a safe and efficacious treatment alternative needs to be established. Phages are nature-assigned scavengers of bacteria, equipped with bacteriolytic enzymes and genetic makeup to parasitize bacterial replication machinery to use against bacteria itself. Several studies have been done on the safety of the bacteriophages, and it has been reported that the phages are safe to use. Most importantly, being nature-made brings an advantage of evolutionary modifications, one of the most essential criteria for tackling ever-evolving bacteria. Selection pressure-driven evaluations make phages indispensable antimicrobial agents and a never-ending arsenal against bacteria.

## 5. Conclusions

With the fast-emerging antibiotic drug resistance, a translatable alternative to antibiotics is of high priority. The biofilm formation by the bacteria further add to the clinical complications, which are inherently recalcitrant to the antibiotics. Bacteriophages, the nature-tailored bacterial scavengers, have emerged as promising alternatives to combat infections due to antibiotic-resistant bacteria. For the first time, we report the antibacterial properties of a novel *Casjensviridae* phage, SAKp02. Our findings demonstrate that phage SAKp02 exhibits potent antibiofilm activity against drug-resistant *K. pneumoniae* clinical isolates. SAKp02 could disrupt the biofilm architecture and lyse the biofilm-residing bacteria within a few hours post-treatment. Our study advocates phages as one of the most effective alternatives to antibiotics to treat drug-resistant bacterial infections.

## Data Availability

The original contributions presented in the study are included in the article/Supplementary Material, further inquiries can be directed to the corresponding author/s.

## Supplementary Materials

The following supporting information can be downloaded at: https://www.mdpi.com/article/10.3390/pharmaceutics16070904/s1, Table S1: Predicted ORFs in SAKp02 genome.

## Abbreviations

ESKAPE	*Enterococcus faecium*, *Staphylococcus aureus*, *Klebsiella pneumoniae*, *Acinetobacter baumannii*, *Pseudomonas aeruginosa*, and *Enterobacter* spp.
WHO	World Health Organization
MMMH	Madras Medical Mission Hospital
ORF	Open Reading Frame
CDS	Coding Sequence
cKp	Classic *K. pneumoniae*
hyKp	Hypervirulent *K. pneumoniae*
EPS	Extracellular Polymeric Substance
BP	Base Pair
ESBL	Extended-Spectrum Beta-lactamase
MDR	Multi Drug Resistance
XDR	Extremely Drug Resistance
TEM	Transmission Electron Microscopy
SEM	Scanning Electron Microscopy
OD	Optical Density
