# Supplementary material for: A New Casjensviridae Bacteriophage Isolated from Hospital Sewage for Inactivation of Biofilms of Carbapenem Resistant Klebsiella pneumoniae Clinical Isolates
Source: Pharmaceutics. 2024 Jul 5;16(7):904. doi: 10.3390/pharmaceutics16070904 (PMC11280391; doi:10.3390/pharmaceutics16070904)
Supplement: Supplementary file 1 [file pharmaceutics-16-00904-s001.zip › pharmaceutics-3053285-supplementary.pdf]

Supplementary Table

Table1: Predicted ORFs in SAKp02 genome.

Sr.	Start	Stop	Size(nt)	Strand	Function
1	1665	1	1664	-	Phage head, portal protein B
2	1916	1665	251	-	Phage head, head-tail joining protein W
3	4003	1928	2075	-	Phage head, terminase DNA packaging protein A
4	4562	3993	569	-	Phage terminase, small subunit
5	6087	4546	1541	-	DNA helicase
6	6376	6080	296	-	VRR-NUC domain-containing protein
7	8418	6376	2042	-	DNA polymerase I
8	9105	8485	620	-	Gp2.5-like ssDNA binding protein and ssDNA annealing protein
9	10501	9152	1349	-	Exonuclease
10	10844	10494	350	-	Cas 4 like protein
11	11291	10899	392	-	Hypothetical protein
12	11344	11481	137	+	Hypothetical protein
13	11521	11802	281	+	Hypothetical protein
14	11799	14390	2591	+	dsDNA helicase
15	14793	15005	212	+	Hypothetical protein
16	15860	15531	329	-	Hypothetical protein
17	16063	15857	206	-	Hypothetical protein
18	16326	16075	251	-	Hypothetical protein
19	17131	16316	815	-	DUF905 domain-containing protein
20	17510	17298	212	-	Hypothetical protein
21	17833	17507	326	-	Hypothetical protein
22	18472	17843	629	-	Hypothetical protein
23	18840	18475	365	-	Hypothetical protein
24	19040	18843	197	-	MC1-binding protein
25	19477	19040	437	-	Hypothetical protein
26	19714	19490	224	-	Hypothetical protein
27	19976	19848	128	-	Hypothetical protein
28	20158	20535	377	+	Hypothetical protein
29	20595	21044	449	+	Hypothetical protein
30	21022	21366	344	+	Hypothetical protein
31	21379	21681	302	+	Hypothetical protein
32	21674	21907	233	+	Hypothetical protein
33	21904	22218	314	+	Hypothetical protein
34	22215	22547	332	+	Hypothetical protein
35	22537	23607	1070	+	Exonuclease
36	23609	23836	227	+	Tail fiber protein
37	23838	24110	272	+	Endolysin
38	24126	24242	116	+	Hypothetical protein
39	24266	25033	767	+	Hypothetical protein
40	25030	25437	407	+	Hypothetical protein
41	25427	25936	509	+	Hypothetical protein
42	25942	26262	320	+	Hypothetical protein
43	26255	26965	710	+	Putative DNA adenine methylase

44	26968	27183	215	+	Hypothetical protein
45	27187	27450	263	+	Hypothetical protein
46	27428	27820	392	+	Hypothetical protein
47	27855	27980	125	+	Hypothetical protein
48	27958	29142	1184	+	Putative C-specific methylase
49	29203	30630	1427	+	Hypothetical protein
50	30630	31493	863	+	Putative head morphogenesis protein
51	31495	31815	320	+	Hypothetical protein
52	31812	32546	734	+	3'-5' exoribonuclease of *K. pneumonaie*
53	32621	33133	512	+	Hypothetical protein
54	33137	33484	347	+	Hypothetical protein
55	33487	33696	209	+	Hypothetical protein
56	33698	34381	683	+	Hypothetical protein
57	34365	34664	299	+	Hypothetical protein
58	34661	35404	743	+	Nucleoside 2-deoxyribosyltransferase
59	35394	35594	200	+	Signal peptide containing protein
60	35881	35618	263	-	O-Spanin
61	36267	35878	389	-	Hypothetical protein
62	36851	36267	584	-	Phage endolysin
63	37196	36864	332	-	Phage holin
64	37615	37196	419	-	Hypothetical protein
65	41762	37695	4067	-	Hypothetical protein
66	42560	41781	779	-	Hypothetical protein
67	44839	42563	2276	-	Vrion structural protein
68	45051	44839	212	-	Hypothetical protein
69	45278	45051	227	-	Hypothetical protein
70	46139	45288	851	-	Hypothetical protein
71	48969	46144	2825	-	Hypothetical protein
72	53274	48973	4301	-	Hypothetical protein
73	53380	53267	113	-	Tail length tape measure protein
74	53964	53506	458	-	Tail assembly chaperone
75	55210	54071	1139	-	Major tail protein
76	55727	55224	503	-	Tail terminator
77	56344	55724	620	-	Phage tail, component Z
78	56709	56344	365	-	Tail attachment protein
79	56980	56711	269	-	Hypothetical protein
80	58095	57031	1064	-	Phage head, major capsid protein E
81	58497	58108	389	-	Phage head, head-DNA stabilization protein D
82	59343	58510	833	-	Phage head, head-tail preconnector protease C
